# The necessity of a more aggressive initial surgical treatment in patients with mesothelioma of the testicular tunica vaginalis

**DOI:** 10.1016/j.amsu.2019.10.012

**Published:** 2019-10-12

**Authors:** Clement Brun, Sophie Giusiano, Khady Thiam, Julien Guinde, Marios Froudarakis, Philippe Astoul

**Affiliations:** a Service de Pneumologie et d’Oncologie Thoracique, Hôpital Nord, Centre Hospitalier Universitaire de Saint Etienne, France; b Service d'Anatomie et Cytologie Pathologiques, Centre Hospitalier Universitaire Nord - Centre de Recherche en Cancérologie de Marseille (CRCM), INSERM U1068/CNRS U7258, Aix-Marseille Université, Marseille, France; c Service de Pneumo-Oncologie, CHU Fann, Université Cheikh Anta Diop, Dakar, Senegal; d Service d’Oncologie Thoracique, Maladies de la Plèvre et Pneumologie Interventionnelle, Centre Hospitalier Universitaire Nord, and, Aix-Marseille University, Marseille, France; e Aix-Marseille University, Marseille, France

**Keywords:** Mesothelioma, Tunica vaginalis, Surgery, Lymph node

## Abstract

Mesothelioma of the tunica vaginalis of the testis (MTVM) is a rare tumor encountering for less than 1% of mesothelioma. Patients suffering from these tumors have poor survival due to local and distant metastasis despite treatment. Actually, no specific treatment recommendations exist for this tumor, yet radical orchidectomy is the gold standard in limited disease. We herein report the case of a 71 patient with MTVM who underwent radical orchidectomy without inguinal lymph node dissection and recurred 2 years later with metastasis in pelvic and mediastinal lymph nodes. Despite systemic chemotherapy combining pemetrexed, bevacizumab and Cisplatinum, the disease relapsed eight months later with multiple metastatic lung nodules leading to a treatment shift. We believe that systematic inguinal-iliac lymph node resection should be included in the initial treatment of this tumor.

## Highlights

• Mesothelioma of the testicular tunica vaginalis.
• Actually, no specific treatment recommendations exist for this tumor.
• Current local treatment: orchidectomy without systematic inguinal lymph node dissection.
• Our suggestion for local treatment: orchidectomy with systematic inguinal lymph node dissection.

## 1 Introduction

Mesothelioma of the tunica vaginalis of the testis (MTVT) is a rare localization accounting for less than 1% of mesotheliomas [1]. It is an aggressive neoplasm [2] that can be induced by asbestos exposure [3]. Currently, no recommendations for specific treatment exist for this tumor and it is important to report all these rare cases to establish evidence-based treatments.

We present a case of a 71-year old male with hydrocele that was subsequently diagnosed with a MTVT. He underwent radical inguinal orchidectomy and relapsed 2 years later with mediastinal and retroperitoneal lymph-node metastasis and presented a final evolution with multiple metastatic lung nodules. Our case report is in line with the SCARE criteria [4] and the patient gave a consent for publication.

## 2 Case report

A 71-year-old male was seen at a routine prostate gland consultation. On clinical examination a large hydrocele was noted. Testicular ultrasound showed a right hydrocele with the presence of echogenic material in favor of chronicity and a 3 cm heterogeneous testicular tumor, located on the antero-inferior wall, hyper-vascularized in doppler. The contralateral testis was normal.

**Fig. 1:**
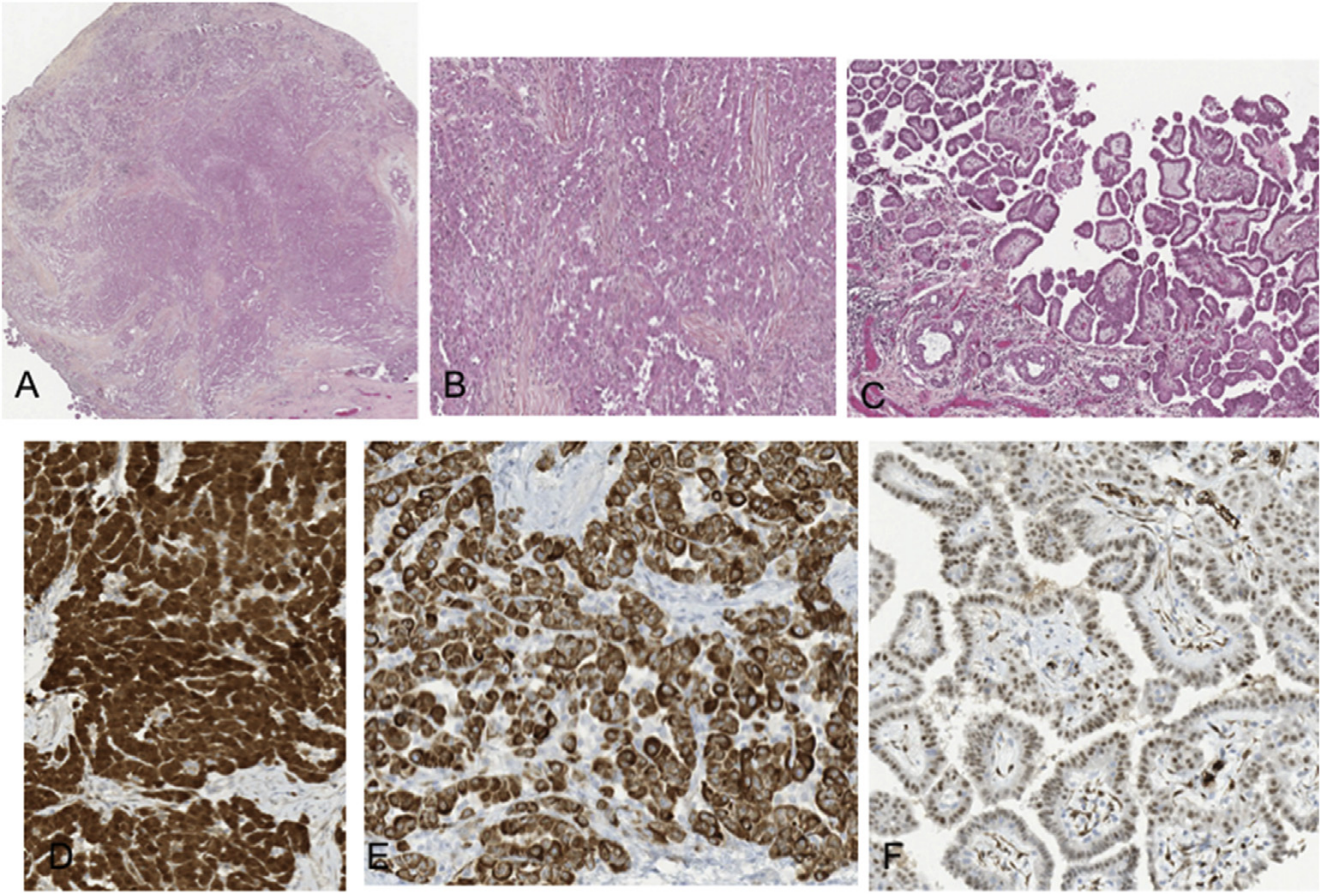
Epithelioid mesothelioma infiltrating the tunical vaginalis of the testis. A. nodular epithelial proliferation invading fibrous tissue (HEx25) - B. moderately atypical tumor cells forming tubulopapillary structures (HEx100) - C. tumor cells forming papillary structures (HEx100) - D. strong nuclear and cytoplasmic expression of calretinine (DABx200) - E. CK5-6 expression (DAB x 200) – F. WT1 nuclear expression (DABx200). HE: Hematoxylin Eosin. DAB: 3,3′ diaminobenzidine.

His history included 20 pack-years of smoking, arterial hypertension, sleep apnea syndrome treated by CPAP since the age of 55, a primary tuberculous infection in childhood with a sequelae of pulmonary subpleural nodule, an idiopathic sideroblastic anemia under erythropoietin since 2012, a treated hypothyroidism as well as dyslipidemia. He did not exhibit exposure to asbestos in his laboris or domestic curriculum. The patient was in excellent shape. Thoracic auscultation was normal. Tumor markers such as total human chorionic gonadotrophin (HCG) and α-fetoprotein (α-FP) were normal. Thoraco-abdomino-pelvic computed tomography (CT) showed no metastasis. Surgical exploration revealed many hard and fleshy nodules on the testicular vaginalis. A radical orchidectomy was performed without lymph node excision. Histology noted a malignant mesothelioma of epithelioid type consisting of papillary and tubular structures. The tumor expressed the typical mesothelial markers of Calretinin, CK 5/6 and WT1 (Fig. 1). Limits were healthy. Postoperative positron emission tomography combined to computed tomography (PET-CT) showed the absence of metastasis. The patient was followed with PET-CT every three months without any further treatment.

**Fig. 2:**
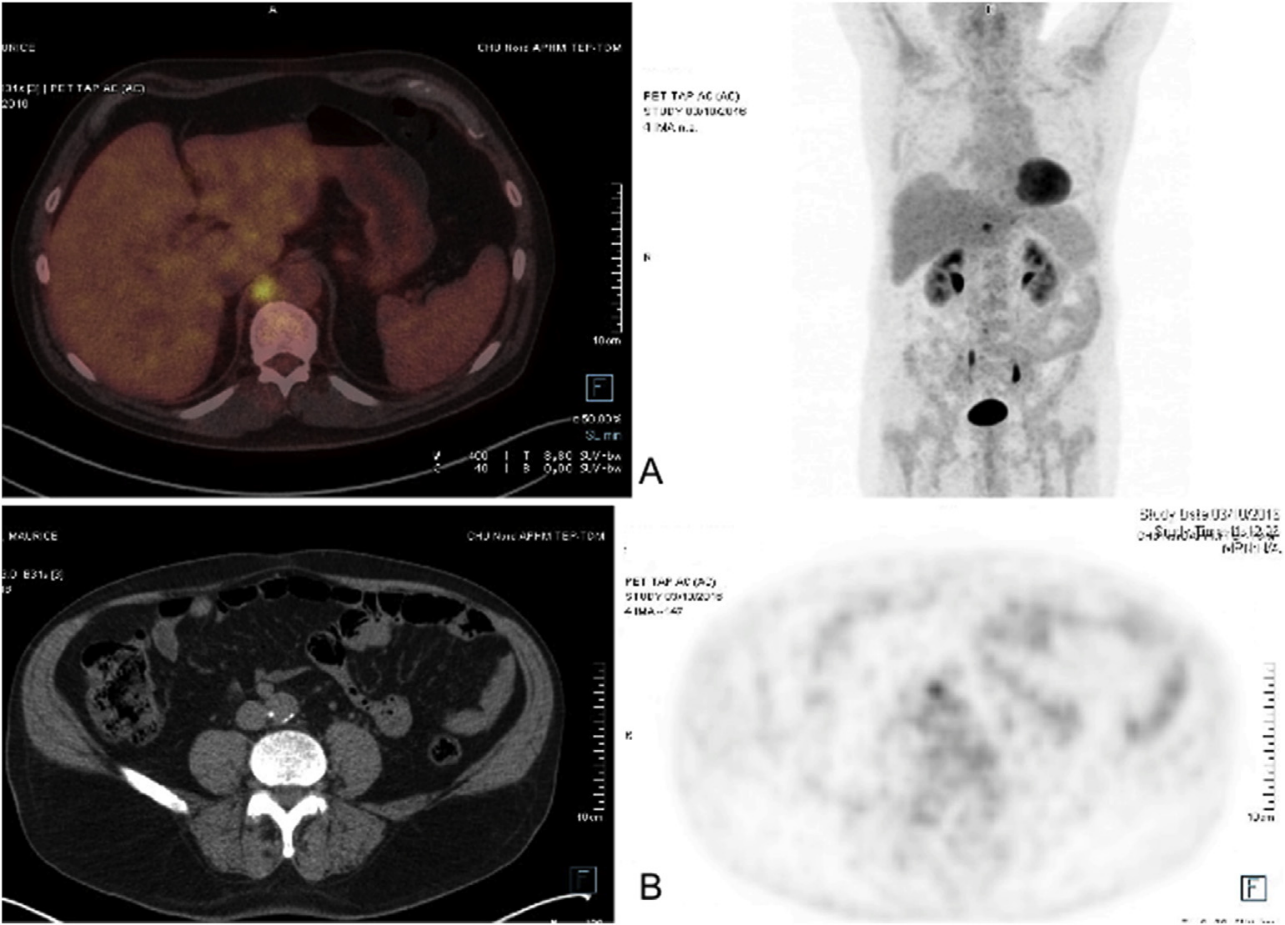
PET-CT at 24 months post-operatively showing: A. lower mediastinal hypermetabolic lymphadenopathy - B. a less hypermetabolic retroperitoneal lymphadenopathy.

**Fig. 3:**
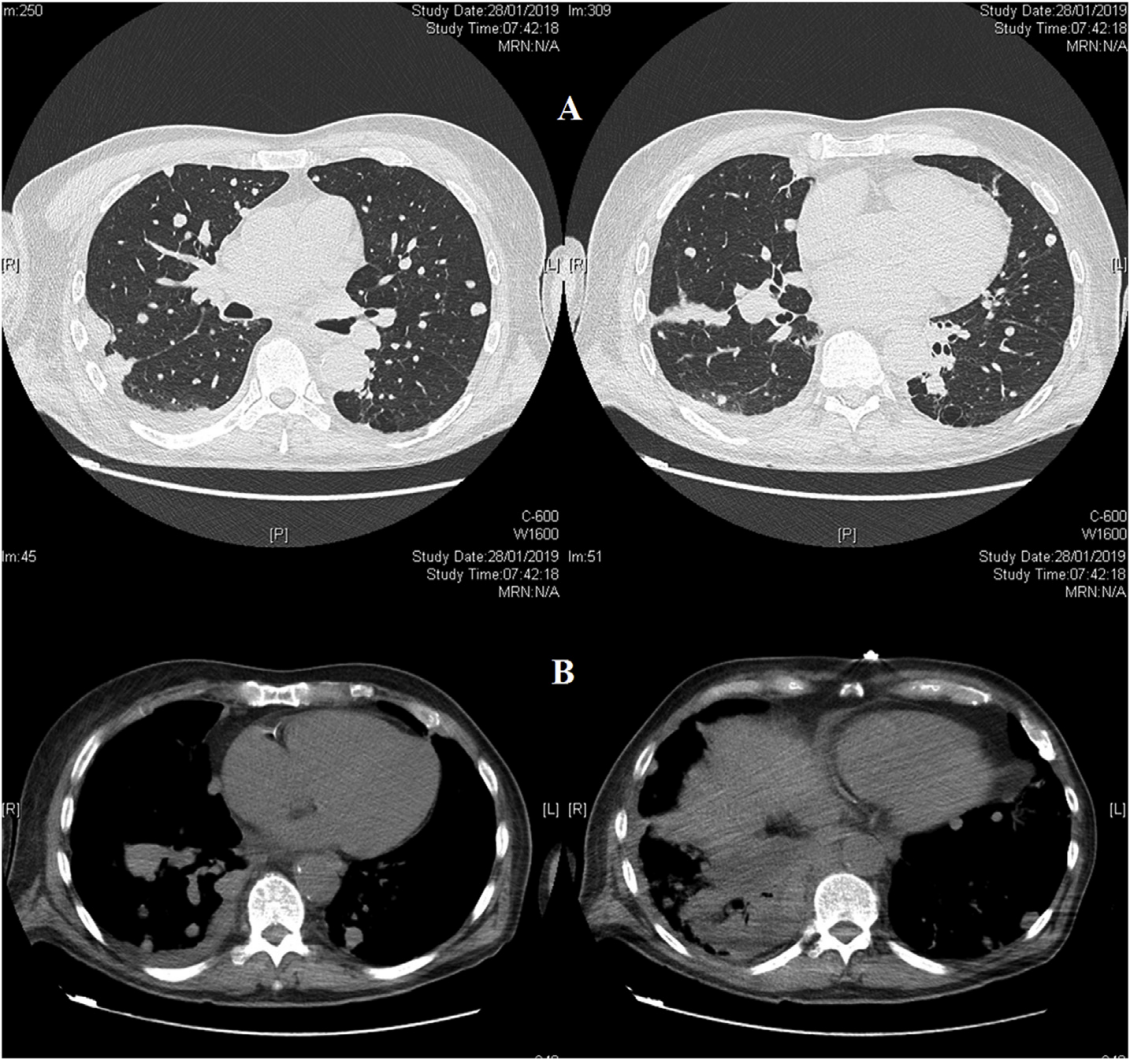
Patient's CT scan with multiple metastatic lung nodules (A) and irregular pleural thickening.

Twenty four months after initial treatment, CT showed a 14 mm lower mediastinal lymphadenopathy, which was hypermetabolic (SUV max at 8.7) on PET (Fig. 2A) associated to a hypermetabolism (SUV 4.8) of a retroperitoneal lymphadenopathy (Fig. 2B). A trans-parietal CT-guided biopsy of the mediastinal node was decided and histology showed mesothelioma relapse. Chemotherapy was then undertaken combining platinum with pemetrexed and bevacizumab. After six cycles, the evaluation showed a stable disease and a follow-up was decided. Eight months later, the disease relapsed with multiple metastatic lung nodules (Fig. 3).

## 3 Discussion

The standardized incidence of MTVT is actually 0.2 cases per million inhabitants in Italy concerning patients over 65 years old in 70% of the cases [5]. Asbestos exposure has been recognized as the main risk factor for MTVT with significant latency (25 years in the average) between exposure and diagnosis [3,5]. The mechanism of involvement of the tunica vaginalis by asbestos fibers remains unclear. However, the tunica vaginalis shares the same embryological origin of pleura, peritoneum and pericardium. It has been proposed that a lymphatic route or the bloodstream may mediate the distribution of asbestos fibers from the lung to other organs [3]. However, up to half of the published cases have not a documented exposure to asbestos, as in our patient [3].

The main presentation in MTVT is hydrocele in 60% of the cases, yet others are possible such as spermatic cord torsion, scrotal hernia or testicular mass [3]. Ultrasound is the preferred examination in the screening of testicular tumors. It may reveal testicular vaginalis thickening, multiple hyper vascularized nodules and heterogeneous material in the hydrocele [6]. Histology and occasionally cytology provides diagnosis [6]. Histopathologically the tumor is usually an epithelial mesothelioma with papillary, tubulopapillary or solid pattern, yet a spindle-cells denoting the sarcomatoid subtype or a biphasic pattern may occure [[6], [7], [8]]. Immunohistochemistry is indicative of mesothelioma with an expression of calretinin, WT-1, CK-5/6. Differential diagnosis from other testicular tumors such as rete testis carcinoma, epididymis cystadenocarcinoma and cancer metastasis of lung, kidney, prostate, stomach and colon is important [7,8].

Radical orchidectomy is the treatment of choice with a median overall survival of 24 months. The disease recurs in 60% of cases within 2 years after radical orchidectomy, with a median survival of 12 months after relapse. Although authors propose initial inguinal or pelvic dissection when malignant mesothelioma is suspected, this intervention is still under discussion because of the low incidence of lymph node metastasis at diagnosis. Carp and associates [9] suggested that a retroperitoneal lymph node dissection should be performed after the pelvic nodes are positive. In a similar case Smith and collaborators [10] suggested that pelvic or retroperitoneal lymph nodes dissection should be included in the definitive treatment as well as in the staging of this tumor. Also, Gupta and collaborators in their report of two cases propose to systematically dissect the pelvic nodes after CT staging and in case of positivity a retroperitoneal node dissection should follow [11].

Mesothelioma of the tunica vaginalis is an aggressive tumor as is of the thoracic and abdominal localisations. Therefore, in such a case in addition to total orchidectomy, consideration should be given to lymph node dissection (inguinal, pelvic and retroperitoneal) at initial treatment as these are the most common sites of metastasis [2]. However, confusion for a less aggressive treatment may be related to the fact that the different series report data from mixed patients, with MTVT, reactive mesothelial hyperplasia (RMH) and well differentiated papillary mesothelioma (WDPM) [2,12,13]. Some of these MTVT mixed with these entities relapse during surveillance and therefore may be overlooked initially, as suggested by Jones [2]. Indeed, no problem exists to recognize the mesothelioma when there is histological evidence of infiltration but WDPM with minimal underlying invasion of the tunica may be difficult to distinguish from RMH [2,8]. Both the clinical presentation and histological features are helpful. Typically RMH is a microscopic finding involving a hernia sac distinctly different from the presence of a mass with the characteristic studding of a hydrocele sac. Microscopic examination of a WDPM reveals complex arborizing papillary fibrous stalks, which are absent in RMH [2].

Our patient had a close post-operative surveillance as recommended by the possibility of relapse [2]. The surveillance should include CT and ultrasound every 3 months for 2 years and then every 6 months for further 3 years [3]. We have added PET to our surveillance to detect metastasis, although no data exists in the literature for MTVT. By this approach, we were able to identify mediastinal and peritoneal lymph node metastasis. Furthermore, chemotherapy proposed in our patient after relapse was based on the recommendations for malignant mesothelioma [14].

To conclude, our patient with MTVT relapsed at the nodal level 2 years after the initial treatment. We recommend a more aggressive approach by adding to the radical orchidectomy a systematic lymph node dissection in this patient population. We also recommend the use of PET-CT in the post operative surveillance. This also points out the necessity to establish recommendations for rare localizations of mesothelioma. Because of the rarity of the neoplasm and its poor prognosis, the need of a large prospective multicenter study, enrolling all cases of MTVT is important for accurate management of this uncommon, but aggressive tumor.

## Ethical approval

Not applicable to the article (case report).

## Sources of funding

All authors declare no funding source in relation to the manuscript.

## Author contribution

All authors contributed equally for this article.

## Guarantor

I am the guarantor of this article.

Pr. Marios E. Froudarakis.

Department of Pneumonology and Thoracic Oncology.

University Hospital of Saint-Etienne.

42100 Saint-Etienne, France.

## Consent

The authors have obtained the written and signed consent from the patient to publish this case which is available upon request (see also last sentence in the introduction and at the end of the manuscript just after references).

## Registration of research studies

Not applicable to the article (case report).

## Provenance and peer review

Not commissioned, externally peer reviewed.

## Declaration of competing interest

All authors state that they have no financial or personal relationship with other people or organisations that could inappropriately influence (bias) this work.

## References

[1] Barbera V., Rubino M. (1957). Papillary mesothelioma of the tunica vaginalis. Cancer.

[2] Jones M.A., Young R.H., Scully R.E. (1995). Malignant mesothelioma of the tunica vaginalis. A clinicopathologic analysis of 11 cases with review of the literature. Am. J. Surg. Pathol..

[3] Mensi C., Pellegatta M., Sieno C., Consonni D., Riboldi L., Bertazzi P.A. (2012). Mesothelioma of tunica vaginalis testis and asbestos exposure. BJU Int..

[4] Agha Riaz A., Borrelli Mimi R., Farwana Reem, Koshy Kiron, Fowler Alexander J., Orgill Dennis P. (2018). For the SCARE Group. The SCARE 2018 statement: updating consensus Surgical CAse REport (SCARE) guidelines. Int. J. Surg..

[5] Marinaccio A., Binazzi A., Di Marzio D., Scarselli A., Verardo M., Mirabelli D., Gennaro V., Mensi C., Merler E., De Zotti R., Mangone L., Chellini E., Pascucci C., Ascoli V., Menegozzo S., Cavone D., Cauzillo G., Nicita C., Melis M., Iavicoli S. (2010). Incidence of extrapleural malignant mesothelioma and asbestos exposure, from the Italian national register. Occup. Environ. Med..

[6] Bertolotto M., Boulay-Coletta I., Butini R., Dudea S.M., Grenier N., Oltmanns G., Ramchandani P., Stein M.W., Valentino M., Derchi L.E. (2016). Imaging of mesothelioma of tunica vaginalis testis. Eur. Radiol..

[7] Karpathiou G., Stefanou D., Froudarakis M.E. (2015). Pleural neoplastic pathology. Respir. Med..

[8] Attanoos R.L., Gibbs A.R. (2000). Primary malignant gonadal mesotheliomas and asbestos. Histopathology.

[9] Carp N.Z., Petersen R.O., Kusiak J.F., Greenberg R.E. (1990). Malignant mesothelioma of the tunica vaginalis testis. J. Urol..

[10] Smith J.J., Malone M.J., Geffin J., Silverman M.L., Libertino J.A. (1990). Retroperitoneal lymph node dissection in malignant mesothelioma of tunica vaginalis testis. J. Urol..

[11] Gupta N.P., Agrawal A.K., Sood S., Hemal A.K., Nair M. (1999). Malignant mesothelioma of the tunica vaginalis testis: a report of two cases and review of literature. J. Surg. Oncol..

[12] Segura-Gonzalez M., Urias-Rocha J., Castelan-Pedraza J. (2015). Malignant mesothelioma of the tunica vaginalis: a rare neoplasm--case report and literature review. Clin. Genitourin. Cancer.

[13] Brimo F., Illei P.B., Epstein J.I. (2010). Mesothelioma of the tunica vaginalis: a series of eight cases with uncertain malignant potential. Mod. Pathol..

[14] Zauderer M.G. (2016). A new standard for malignant pleural mesothelioma. Lancet.

